# Simultaneous Estimation of Two Coupled Hydrogen Bond Geometries from Pairs of Entangled NMR Parameters: The Test Case of 4-Hydroxypyridine Anion

**DOI:** 10.3390/molecules27123923

**Published:** 2022-06-18

**Authors:** Elena Yu. Tupikina, Mark V. Sigalov, Peter M. Tolstoy

**Affiliations:** 1 Institute of Chemistry, St. Petersburg State University, 199034 St. Petersburg, Russia; peter.tolstoy@spbu.ru; 2 Department of Chemistry, Ben-Gurion University of the Negev, Beer-Sheva 84105, Israel; msigalov@bgu.ac.il

**Keywords:** hydrogen bonds, NMR, structure determination

## Abstract

The computational method for estimating the geometry of two coupled hydrogen bonds with geometries close to linear using a pair of spectral NMR parameters was proposed. The method was developed based on the quantum-chemical investigation of 61 complexes with two hydrogen bonds formed by oxygen and nitrogen atoms of the 4-hydroxypyridine anion with OH groups of substituted methanols. The main idea of the method is as follows: from the NMR chemical shifts of nuclei of atoms forming the 4-hydroxylpyridine anion, we select such pairs, whose values can be used for simultaneous determination of the geometry of two hydrogen bonds, despite the fact that every NMR parameter is sensitive to the geometry of each of the hydrogen bonds. For these parameters, two-dimensional maps of dependencies of NMR chemical shifts on interatomic distances in two hydrogen bonds were constructed. It is shown that, in addition to chemical shifts of the nitrogen atom and quaternary carbon, which are experimentally difficult to obtain, chemical shifts of the carbons and protons of the CH groups can be used. The performance of the proposed method was evaluated computationally as well on three additional complexes with substituted alcohols. It was found that, for all considered cases, hydrogen bond geometries estimated using two-dimensional correlations differed from those directly calculated by quantum-chemical methods by not more than 0.04 Å.

## 1. Introduction

Spectral NMR diagnostics is an important tool for studies of complexes with hydrogen bonds, as oftentimes it is the main way to obtain reliable information about hydrogen bond geometry and strength [1,2,3,4,5,6,7]. At the moment, there are a number of correlational equations linking the numerical value of spectral NMR parameters and the geometry/energy of a single hydrogen bond [8]. Some of them are well established and widely used, and their applicability was tested for multiple set of complexes, while others are applicable only to particular complexes and conditions. Among the parameters whose change upon the formation of a hydrogen-bonded complex is correlated with the strength of a X–H···Y hydrogen bond, one can distinguish two major groups. The parameters of the first group are characterized by a monotonous change along the proton transfer coordinate. As representatives of this group, one can name the chemical shifts of heavy nuclei *δ*_X_ and *δ*_Y_ and the spin–spin coupling constants ^1^*J*_XH_ and ^1^*J*_HY_ [9,10,11,12,13]. Parameters from the second group exhibit extremal values for the shortest (strongest) hydrogen bond. As an example, one could mention the chemical shift of the bridging proton *δ*_H_ or the spin–spin coupling constant ^2h^*J*_XY_, whose change along the proton transfer coordinate can be characterized by a bell-shaped curve [3,4,5,14,15,16,17]. Parameters from the first group are preferable when solving the inverse spectral problem as their value unequivocally corresponds to a single geometry of a hydrogen bond. For parameters from the second group, there is an uncertainty that is caused by the fact that a single value of the parameter can be observed for two configurations of a hydrogen bond.

Despite the successful development of numerous methods of spectral diagnostics for estimating the geometry and strength of hydrogen bonds, there are some relevant issues that remain to be solved. For example, it is not known how the inverse spectral problem can be solved for systems with multiple mutually interacting hydrogen bonds, since the magnitude of each spectral parameter can be influenced by the presence of all hydrogen bonds simultaneously.

**Figure 1:**
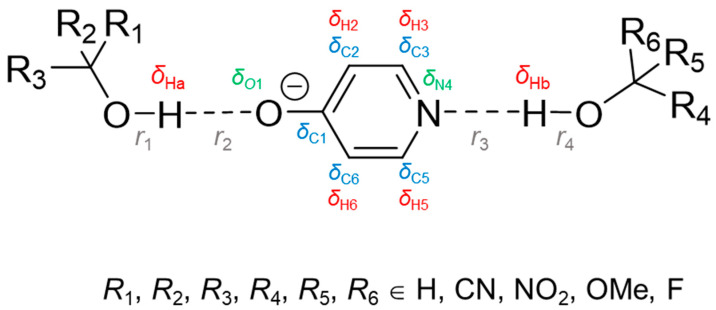
Schematic representation of investigated complexes with two hydrogen bonds formed by 4-hydroxypyridine anion as a hydrogen bond acceptor and two substituted methanols as donors. Geometric parameters (interatomic distances *r*_1_, *r*_2_, *r*_3_ and *r*_4_, grey) and spectral NMR parameters (chemical shifts of bridging protons *δ*_Ha_, *δ*_Hb_, red; atoms of 4-hydroxypyridine anion’s ring: oxygen atom *δ*_O1_, green; carbons *δ*_C1_, *δ*_C2_, *δ*_C3_, *δ*_C5_, *δ*_C6_, blue; hydrogens *δ*_H2_, *δ*_H3_, *δ*_H5_, *δ*_H6_, red, and nitrogen *δ*_N4_, green) considered in this work are indicated.

The aim of this computational work is to demonstrate the possibility of solving the inverse spectral problem for a system with two coupled hydrogen bonds, in particular, to find such pairs of spectral NMR variables that could be used for an unequivocal evaluation of geometries of a pair of mutually influencing hydrogen bonds. As a model system, we consider a 4-hydroxypyridine anion, which can form two hydrogen bonds as a hydrogen bond acceptor (from the oxygen and nitrogen side) with two substituted methanol molecules (Figure 1).

Substitution was made by a subsequent replacement of hydrogen atoms by CN, NO_2_, OMe or F groups. The resulting set of substituted methanols served as a source of proton donors of various strengths, regardless of their practical chemical stability. Table 1 shows the pattern of substitution used within each of the four sub-series of complexes (full list of substituents can be found in Table S1 in the Supporting Information). Together with the unsubstituted complex *R*_1_ = *R*_2_ = *R*_3_ = H, *R*_4_ = *R*_5_ = *R*_6_ = H, 61 complexes with two hydrogen bonds were considered in this work. In such systems, OHO and OHN hydrogen bonds can be of various strengths (from weak to moderate-strong, it is controlled by the choice of substituents). All hydrogen bonds are fairly linear (the range of OHO and OHN angles is 156°–179°, see Table S1) and are formed along the direction of the lone pair localization on proton-accepting O and N atoms. The central 4-hydroxypyridine anion can be negatively charged (OH···O^−^PyrN···HO), neutral (O^−^···HOPyrN···HO or OH···OPyrNH···O^−^) or even positively charged (O^−^···HOPyrNH^+^···O^−^), depending on proton positions in hydrogen bonds. Therefore, such a choice of model systems is suitable for the investigation of complexes with two mutually influencing hydrogen bonds (OHO and OHN) in a wide range of hydrogen bond geometries formed by neutral or charged species. Throughout the work, parameters associated with the OHO hydrogen bond will be denoted with an index “a” and those of OHN—with an index “b”.

**Figure 2:**
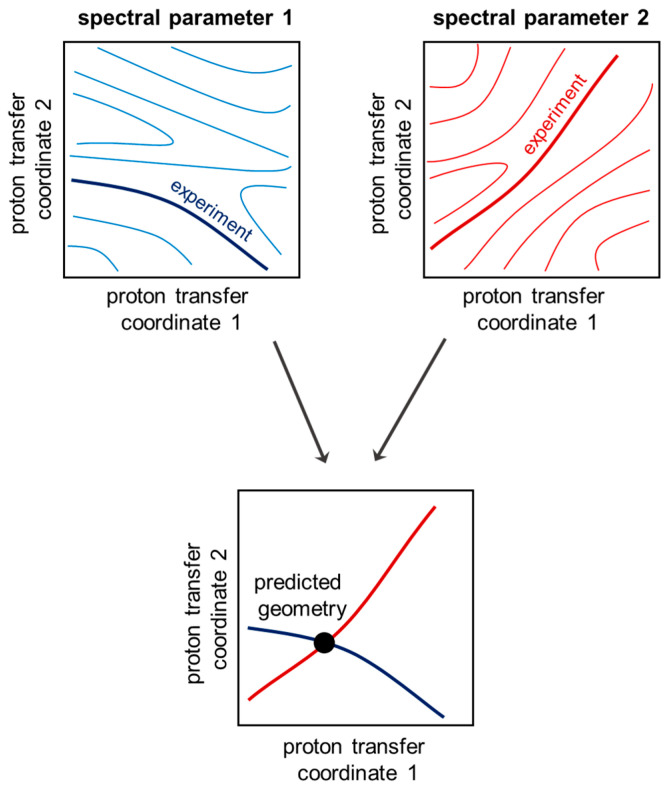
Schematic representation of the algorithm of inverse spectral problem solving. The isolines are drawn arbitrarily and do not correspond to any particular case.

The proposed concept for simultaneous solution of the inverse spectral problem for both coupled hydrogen bonds is as follows. In order to determine the geometries of two hydrogen bonds, two spectral parameters are needed. A particular value of a chosen spectral parameter corresponds to multiple combinations of the two hydrogen bond geometries, forming an isoline on a distribution map of a spectral parameter as a function of two proton transfer coordinates (Figure 2, top). However, if one measures two spectral parameters, the intersection of two isolines (Figure 2, bottom) gives the geometry of the two hydrogen bonds in a coupled system. The case with a single intersection of two isolines is preferable and will be used in the following discussion as the criterion for a choice of spectral parameters for solving the inverse spectral problem.

## 2. Results and Discussion

**Figure 3:**
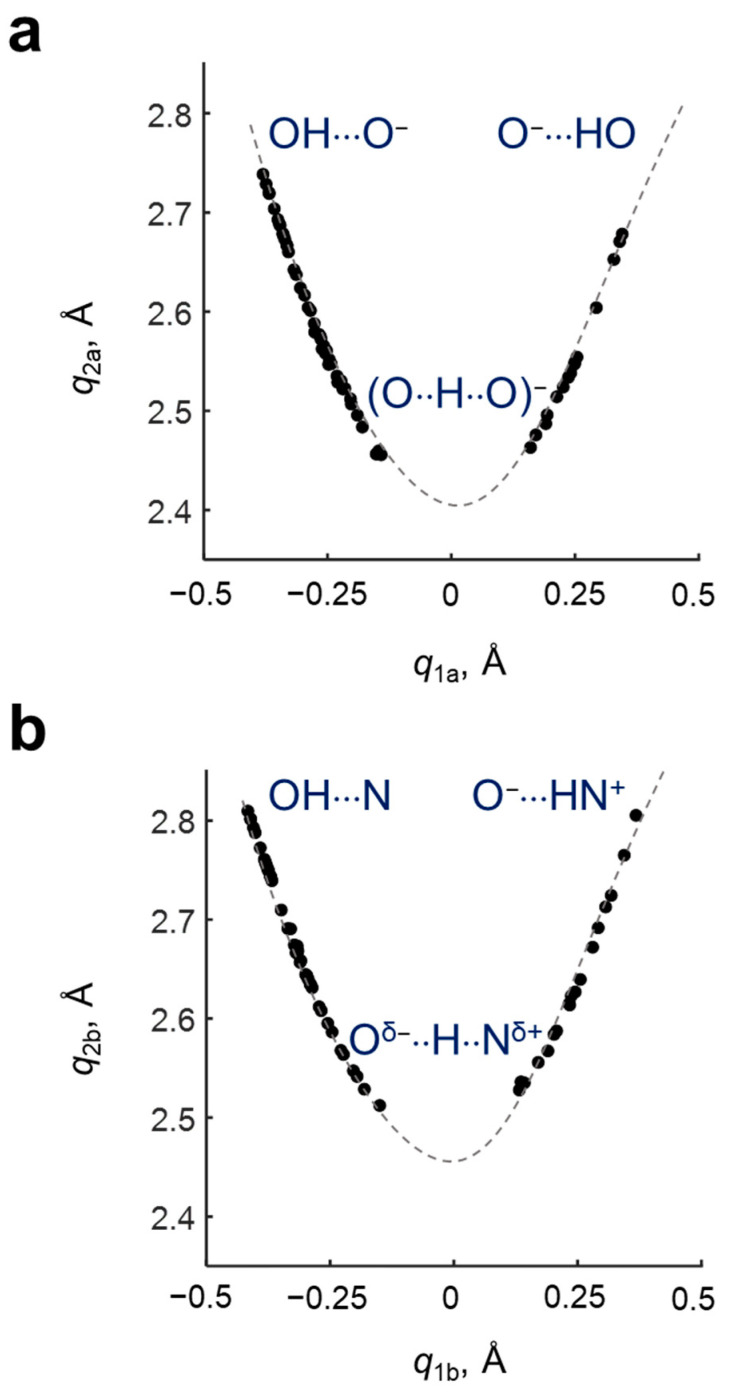
Dependencies of the (**a**) $q_{2a}=r_{1}+r_{2}$ coordinate on $q_{1a}=0.5\cdot \left(r_{1}-r_{2}\right)$ for OHO hydrogen bond; dependencies of the (**b**) $q_{2b}=r_{4}+r_{3}$ coordinate on $q_{1b}=0.5\cdot \left(r_{4}-r_{3}\right)$ for OHN hydrogen bond. Dashed lines are guides for the eye.

The geometric parameters (interatomic distances *r*_1_, *r*_2_, *r*_3_ and *r*_4_) of hydrogen bonds in investigated complexes are collected in Table S1. The set of complexes covers a wide range of hydrogen bonds geometries—there are complexes without proton transfer in both hydrogen bonds (OH···O^−^PyrN···HO), complexes with proton transfer in one of the hydrogen bonds (O^−^···HOPyrN···HO or OH···OPyrNH···O^−^) and complexes with two hydrogen bonds with proton transfer (O^−^···HOPyrNH^+^···O^−^). The dependencies of the $q_{2a}=r_{1}+r_{2}$ coordinate on $q_{1a}=0.5\cdot \left(r_{1}-r_{2}\right)$ for the OHO hydrogen bond and the $q_{2b}=r_{4}+r_{3}$ coordinate on $q_{1b}=0.5\cdot \left(r_{4}-r_{3}\right)$ for the OHN hydrogen bond are shown in Figure 3. It is seen that the complexes cover a range of *q*_1_ from ca. −0.4 to ca. 0.35 Å forming a parabolic curve.

**Figure 4:**
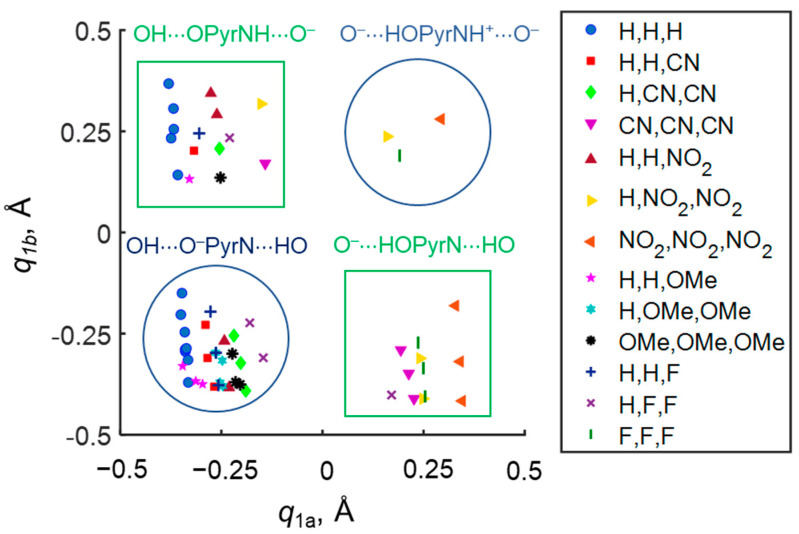
The dependence of proton position in the NH···O hydrogen bond *q*_1b_ on the proton position in the OH···O hydrogen bond *q*_1a_. The shape and color of a marker indicate a series of complexes with the same set of substituents *R*_1_, *R*_2_ and *R*_3_ (shown in legend) and varying set of substituents *R*_4_, *R*_5_ and *R*_6_. Green areas correspond to cooperative hydrogen bonds and blue areas to anti-cooperative ones.

Despite the fact that OHO and OHN hydrogen bonds are divided in space by 4-hydroxypyridine anion, they “feel” the presence of each other through the electronic system of the complex as a whole, the phenomenon is called cooperativity. It manifests as the changes of the proton position in one hydrogen bond depending on the proton position in another hydrogen bond. For example, if one fixes the substituents R_1_, R_2_ and R_3_ in the proximity of the OHO hydrogen bond (see a set of points of the same shape and color in Figure 4), the variation of substituents in OHN hydrogen bond causes a change in *q*_1a_. Complexes with a “tail-to-tail” (OH···O^−^PyrN···HO) and a “head-to-head” (O^−^···HOPyrNH^+^···O^−^) configuration are anti-cooperative (blue areas in Figure 4): i.e., the strengthening of one hydrogen bond causes the weakening of another hydrogen bond. For complexes with “head-to-tail geometry” (O^−^···HOPyrN···HO and OH···OPyrNH···O^−^) (green areas in Figure 4) cooperative effects are observed: i.e., the strengthening of one hydrogen bond causes the strengthening of another. Thus, the character of mutual influence of geometries of OHO and OHN hydrogen bonds in such a system can be either cooperative or anti-cooperative.

**Figure 5:**
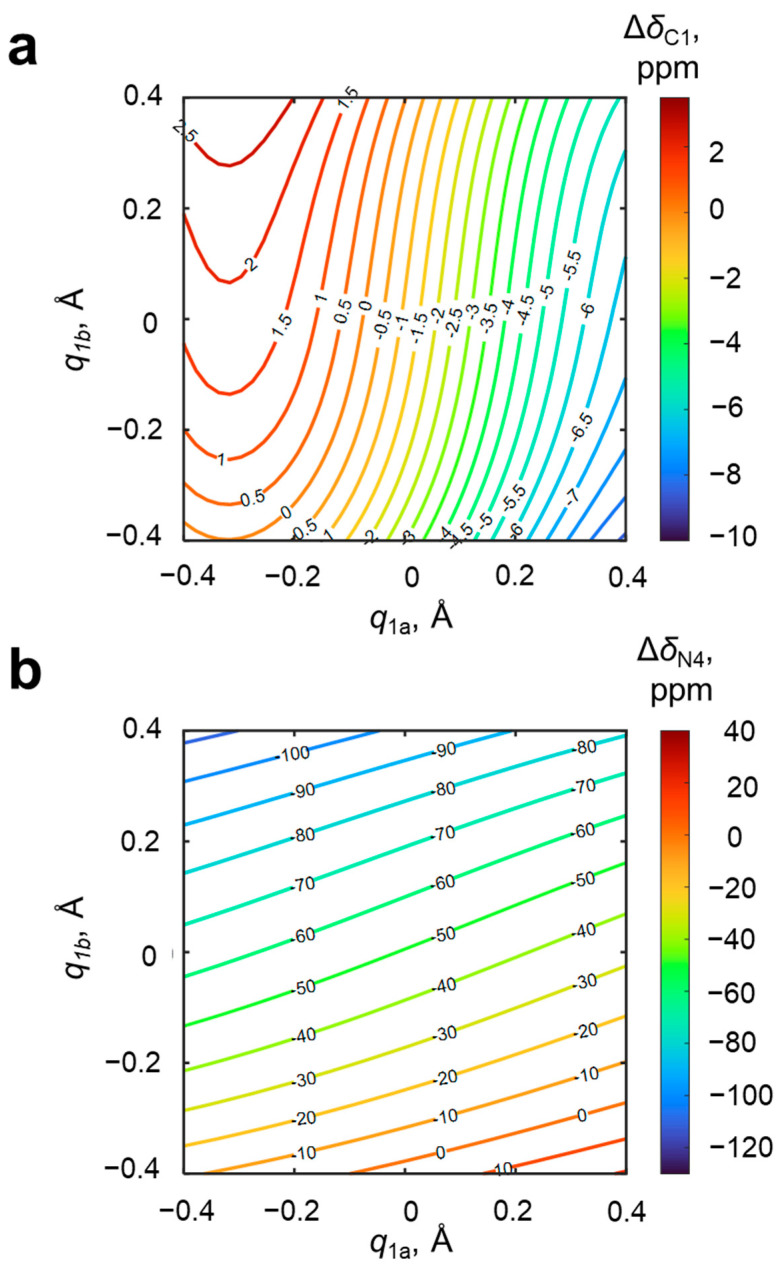
Distributions of the change upon complexation of chemical shift of (**a**) the C1 atom Δ*δ*_C1_ and (**b**) the N4 atom Δ*δ*_N4_ along *q*_1_ coordinates for OHO (*q*_1a_) and OHN (*q*_1b_) hydrogen bonds. The coefficients *a*, *b*_1_, *c*_1_, *d*_1_, *b*_2_, *c*_2_ and *d*_2_ (Equation (1)) and *R*^2^ are given in Table S2. Isolines are drawn with a step of 0.5 and 10 ppm, respectively.

Among the NMR parameters of the 4-hydroxypyridine anion, which potentially are sensitive to the geometries of OH···O and OH···N hydrogen bonds, chemical shifts of the nuclei located in the proximity of hydrogen bonds, i.e., carbon (C1) and nitrogen (N4), are the first candidates. The changes of carbon and nitrogen chemical shifts upon complexation, Δ*δ*_C1_ and Δ*δ*_N4_, are shown in Figure 5.

In Figure 5, it is clearly seen that the C1 carbon chemical shift changes with the change of geometries of both hydrogen bonds—upon moving the bridging proton along OHO hydrogen bond (with fixed geometry of OHN hydrogen bond), Δ*δ*_C1_ changes by up to 8 ppm, and moving it along the OHN hydrogen bond causes a change of Δ*δ*_C1_ by up to 2.5 ppm. In other words, the steepness of the slope of the Δ*δ*_C1_(*q*_1a,_ *q*_1b_) surface is larger in the direction of *q*_1a_ than in the direction of *q*_1b_. Contrarily, the nitrogen chemical shift is more sensitive to the geometry of the closest (OHN) hydrogen bond. The shape of the isolines of the distributions of Δ*δ*_C1_ and Δ*δ*_N4_ makes the unequivocal solving of the inverse spectral problem possible for most cases (except for *q*_1a_ ≈ −0.3 Å due to the shape of the Δ*δ*_C1_ isolines in this region caused by a presence of a hill with its maximal value). Together with the high sensitivity discussed above, this makes the pair Δ*δ*_C1_ and Δ*δ*_N4_ quite promising parameters. However, the experimental issues of measuring nitrogen chemical shift (low natural abundance of ^15^N and line broadening due to quadrupolar interactions in ^14^N NMR spectra) encourage us to discuss additional NMR parameters.

**Figure 6:**
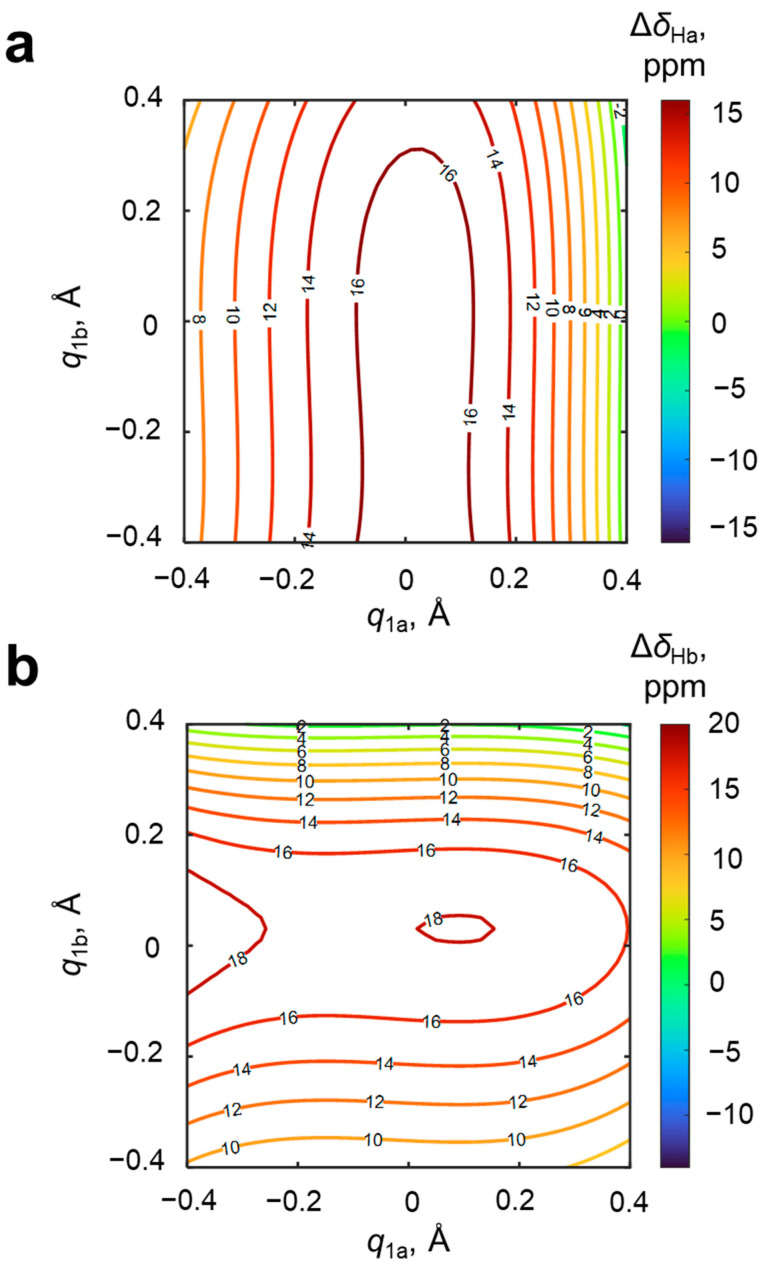
Distributions of the change upon complexation of chemical shift of bridging protons (**a**) in the OH···O hydrogen bond Δ*δ*_Ha_ and (**b**) in the OH···N hydrogen bond Δ*δ*_Hb_ along *q*_1_ coordinates for OHO (*q*_1a_) and OHN (*q*_1b_) hydrogen bonds. The coefficients *a*, *b*_1_, *c*_1_, *d*_1_, *b*_2_, *c*_2_ and *d*_2_ (Equation (1)) and *R*^2^ are given in Table S2. Isolines are drawn with a step of 2 ppm.

The next pair of “promising” NMR parameters are the changes of chemical shifts of bridging protons in the OHO and OHN hydrogen bonds upon complexation, Δ*δ*_Ha_ and Δ*δ*_Hb_; their dependences on *q*_1a_ and *q*_1b_ are shown in Figure 6. Along the OHO hydrogen bond, Δ*δ*_Ha_ increases from 8 to 17 ppm for *q*_1a_ < 0, reaches maximal value at *q*_1a_ ≈ 0 and then decreases to 0 ppm. The change of the geometry of the OHN hydrogen bond (*q*_1b_) does not significantly influence Δ*δ*_Ha_. A similar situation is observed for Δ*δ*_2b_: moving along the OHN hydrogen bond (*q*_1b_) changes Δ*δ*_Hb_ from 9 to 18 ppm for *q*_1b_ ≈ 0 and then down to 0 ppm. It is clearly seen that both surfaces have a hill of maximal values of Δ*δ*_H_ and slopes in the direction of one of *q*_1_ axes (*q*_1a_ for Δ*δ*_Ha_ and *q*_1b_ for Δ*δ*_Hb_). It means that the magnitude of the bridging proton chemical shift in a given hydrogen bond is determined almost exclusively by the geometry and electronic features of the three atoms forming this bond. In other words, the chemical shifts of the two bridging protons are not coupled. For the purposes of solving the reverse spectral problem, this is an advantage, because the magnitude of each chemical shift can be used for the independent evaluation of hydrogen bond geometries.

**Figure 7:**
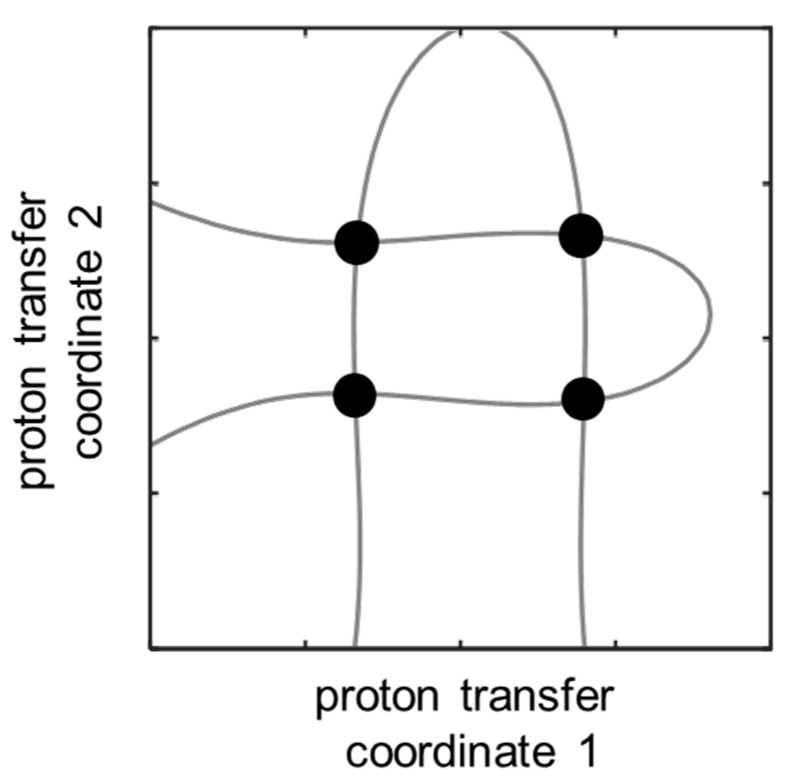
Schematic representation of the non-unequivocal inverse spectral problem solving. The black dots correspond to the four possible solutions. NMR parameters of the 4-hydroxypyridine anion, the chemical shift of carbon *δ*_C2_, *δ*_C3_, *δ*_C5_, *δ*_C6_ and the hydrogen atoms *δ*_H2_, *δ*_H3_, *δ*_H5_, *δ*_H6_, were also analyzed for their sensitivity and applicability for the evaluation of the geometries of OHO and OHN hydrogen bonds. We found that the most promising parameters are arithmetically averaged chemical shifts of C2 and C6 atoms (*δ*_C26_), C3 and C5 atoms (*δ*_C35_) and H3 and H5 atoms (*δ*_H35_). The distributions of changes of these parameters are shown in Figure 8. Both carbon chemical shifts, Δ*δ*_C26_ and Δ*δ*_C35_, are more sensitive to the closest hydrogen bond (OHO and OHN, respectively). The range of values of Δ*δ*_C26_ was about 10 ppm, Δ*δ*_C35_—15 ppm, which makes both of them suitable for the accurate evaluation of hydrogen bond geometries. The change of *δ*_H35_ within the change of the OHO and OHN hydrogen bonds geometries slightly exceeded 1 ppm. The topology of these three surfaces is such that almost all combinations of pair of isolines of Δ*δ*_C26_, Δ*δ*_C35_ and Δ*δ*_H35_ have a single intersection point, thus making the solution of an inverse spectral problem unequivocal.

However, there are two principal problems with such an approach. The first problem was mentioned in introduction and is caused by the fact that a particular value of one of the proton chemical shifts (Δ*δ*_Ha_ or Δ*δ*_Hb_) corresponds to an isoline in distributions shown in Figure 6a,b that form a hairpin curve. Two hairpin isolines have four intersecting points (see Figure 7), i.e., each set of Δ*δ*_Ha_ and Δ*δ*_Hb_ values could correspond to four alternative hydrogen bond geometries. The second problem with using Δ*δ*_Ha_ and Δ*δ*_Hb_ for the evaluation of hydrogen bond geometries is due to the fact that, in the experimental spectrum, it is difficult to distinguish which signal will relate to the OHO and which to the OHN hydrogen bond. This issue demands the usage of additional NMR parameters, for example, chemical shifts of the nuclei of 4-hydroxypyridine.

**Figure 8:**
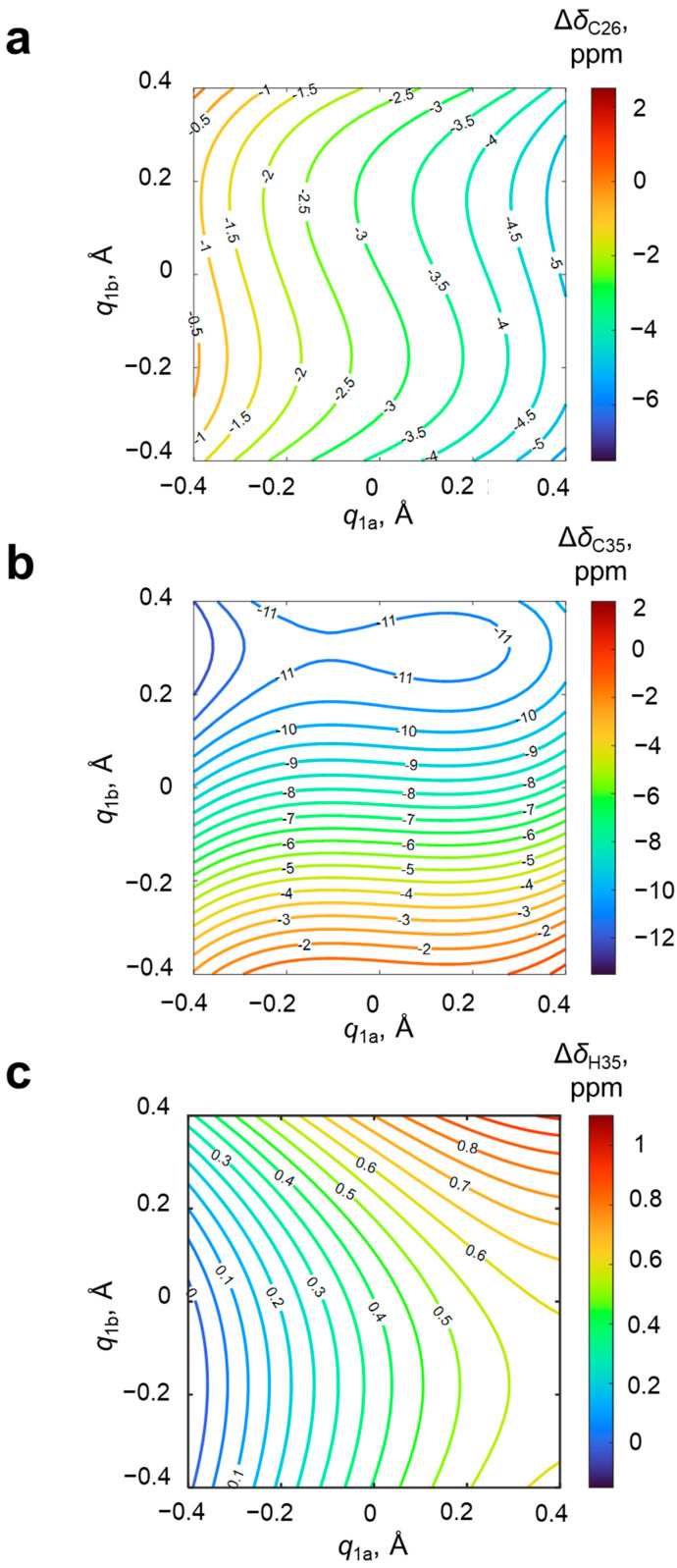
Distributions of the change upon complexation of average chemical shift of (**a**) C2 and C6 atoms Δ*δ*_C26_, (**b**) C3 and C5 atoms Δ*δ*_C35_ and (**c**) H3 and H5 atoms Δ*δ*_H35_ along *q*_1_ coordinates for OHO (*q*_1a_) and OHN (*q*_1b_) hydrogen bonds. The coefficients *a*, *b*_1_, *c*_1_, *d*_1_, *b*_2_, *c*_2_ and *d*_2_ (Equation (1)) and *R*^2^ are given in Table S2. Isolines are drawn with a step of 0.5 ppm for (**a**,**b**) and 0.05 ppm for (**c**), respectively.

For an additional estimation of the mutual influence of OHO and OHN hydrogen bonds, calculations for two extra sets of systems with a single hydrogen bond were performed (4-hydroxypyridine anion with one substituted methanol from either the oxygen or nitrogen side, 10 complexes in each extra set). The results the of NMR calculations (*δ*_C26_ and *δ*_C35_) are presented in Figures S1 and S2 of the Supporting Information. The difference plot between data shown in Figure 8 (system with two coupled hydrogen bonds) and Figures S1 and S2 (hypothetical system with no coupling between hydrogen bond modeled as a sum of systems with a single hydrogen bond) is shown in Figure S3. It is clearly seen that the cooperativity effects on the NMR parameters are substantial (±2–4 ppm, making them non-negligible) and non-monotonous. The strongest effects are observed for the configuration O^−^···HOPyrNH^+^···O^−^.

**Figure 9:**
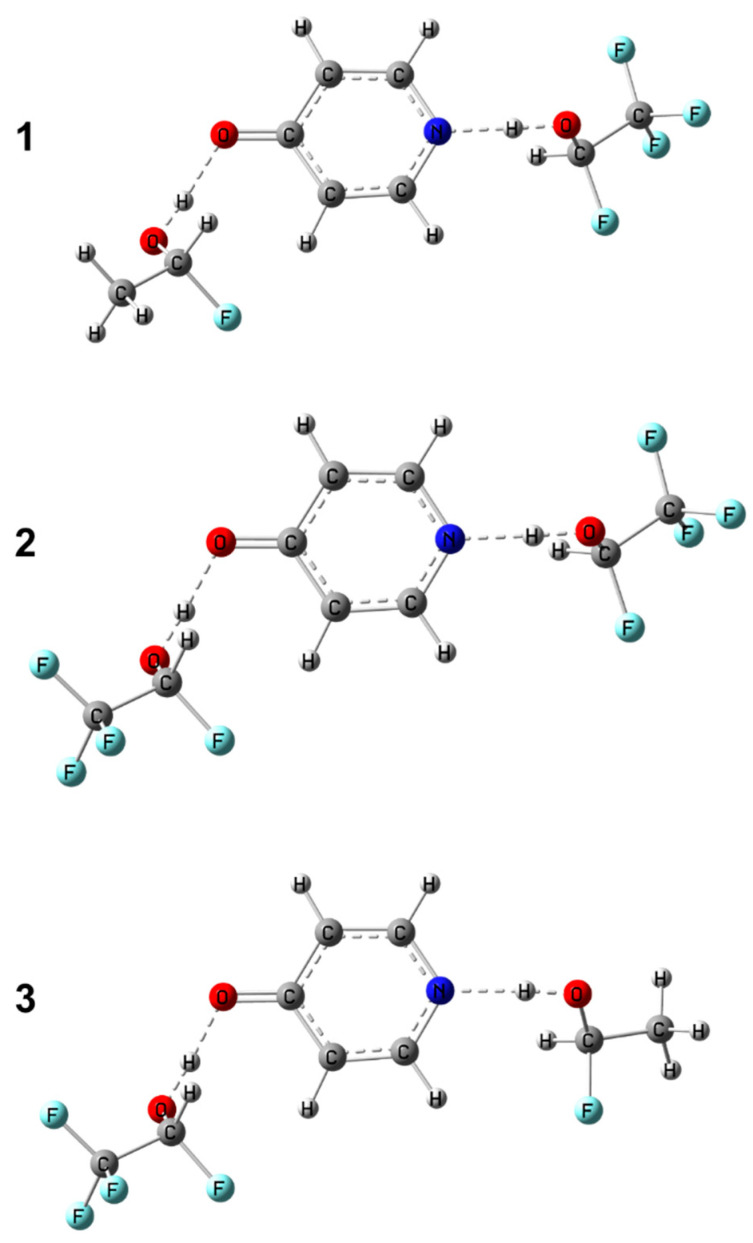
Structures of additional complexes with two hydrogen bonds formed by 4-hydroxypyridine anion as a hydrogen bond acceptor and two substituted methanols as donors used for testing the proposed approach. From top to bottom: **1** (proton donors CH_3_CHFOH, CF_3_CHFOH), **2** (proton donors CH_3_CHFOH, CF_3_CHFOH), **3** (proton donors CF_3_CHFOH, CH_3_CHFOH).

In order to test the approach proposed in this work, we performed additional calculations of structure and NMR parameters for three arbitrarily chosen complexes of 4-hydroxypyridine anion with CH_3_CFHOH and CF_3_CFHOH molecules: **1** (CH_3_CFHOH···^−^OPyrN···HOCFHCF_3_), **2** (CF_3_CFHOH···^−^OPyrN···HOCFHCF_3_) and **3** (CF_3_CFHOH···^−^OPyrN···HOCFHCH_3_); the optimized structures of **1**–**3** are shown in Figure 9. The results of the comparison of the “predicted” geometry based on pairs of NMR chemical shifts and the “real” (calculated) geometries of the two hydrogen bonds are summarized in Table 2 and shown in Figures S4–S6 in the Supporting Information. It can be concluded that hydrogen bond geometries estimated using two-dimensional correlations differ from those directly calculated by quantum-chemical methods by not more than 0.04 Å. The only exception is complex **3** (CF_3_CFHOH and CF_3_CFHOH), for which the chemical shifts of carbons C2 and C6 differ significantly due to the presence of an additional hydrogen bond between the CH-group of the 4-hydroxypyridine anion and the fluorine atom of the substituted methanol (see Figure S7). In this case, using the chemical shift of carbon atom not involved in additional hydrogen bonding instead of the arithmetically averaged chemical shift Δ*δ*_C26_ is more appropriate.

## 3. Computational Details

Geometry optimization was performed using second-order Moller–Plesset perturbation theory (MP2) [12,13] with Dunning’ correlation-consistent polarized double-ζ basis set with diffuse functions aug-cc-pVDZ [14]. All calculated geometries were checked for the absence of imaginary frequencies. Chemical shieldings were calculated using DFT (B3LYP) with the augmented polarization consistent triple-ζ basis set aug-pcS-2, which is specially designed for the calculation of shieldings at the DFT level with a high accuracy [18].

Calculations were carried out using the Gaussian16 software. Computational resources were provided by the Computer Center of Saint Petersburg University Research Park (http://www.cc.spbu.ru/, accessed on 1 April 2020).

Visualization was performed in GaussView 6.0 and MATLAB 2021b software packages.

For the description of the geometries of OHO and OHN hydrogen bonds, the parameters *q*_1_ and *q*_2_ were used [2,16,17]: $q_{1}=0.5\cdot \left(r_{OH}-r_{HY}\right)$, $q_{2}=r_{OH}+r_{HY}$, where Y = O, N. The meaning of *q*_1_ and *q*_2_ coordinates is pretty clear for linear hydrogen bonds—the *q*_1_ coordinate is the shift of the hydrogen atom from the hydrogen bond center, the *q*_2_ coordinate is the total length of the hydrogen bond (for strictly linear hydrogen bonds, *q*_2_ is the distance between the heavy atoms O...O or O...N; it should be noted that, as hydrogen bonds deviate from linearity, *q*_2_ loses this geometrical meaning, because in this case *q*_2_ is slightly longer than the distance O...O or O...N). For the OHO hydrogen bond, the following pair of coordinates was used $q_{1a}=0.5\cdot \left(r_{1}-r_{2}\right)$ and $q_{2a}=r_{1}+r_{2}$ and for OHN, they were $q_{1b}=0.5\cdot \left(r_{4}-r_{3}\right)$ and $q_{2b}=r_{4}+r_{3},$ respectively.

The algorithm of construction for the distributions of spectral parameters (Δ*δ*_C1_, Δ*δ*_N4_ and others discussed in this work) along the $q_{1a}$ and $q_{1b}$ was as follows. For a set of complexes shown in Figure 1, NMR parameters were calculated. Let us denote the spectral parameter as *f*. The value of a change of a spectral parameter upon complexation is defined as $\Delta f=f_{complex}-f_{free}$ (where $f_{free}$ is the value of a given spectral parameter for an isolated 4-hydroxypyridine anion or methanol, and $f_{complex}$ is the value of the same parameter within a hydrogen-bonded complex). Calculated Δ*f* values were approximated as a function of $q_{1a}$ and $q_{1b}$ using the Curve Fitting Tool implemented in the MATLAB 2021b software package by a polynomial function of a third-degree along $q_{1a}$ and $q_{1b}$ without cross-terms:

(1) $$\Delta f\left(q_{1a},q_{2b}\right)=a+b_{1}q_{1a}+c_{1}q_{1a}^{2}+d_{1}q_{1a}^{3}+b_{2}q_{1b}+c_{2}q_{1b}^{2}+d_{2}q_{1b}^{3}$$

The coefficients *a*, *b*_1_, *c*_1_, *d*_1_, *b*_2_, *c*_2_ and *d*_2_ for all discussed parameters and the coefficient of determination *R*^2^ for each approximation are given in Table S2 in the Supporting Information. The resulting functions were plotted as contour plots, in which each isoline corresponds to the particular value of Δ*f* and is marked by a color and an isovalue for clarity in the following figures. The polynomials were taken as one of the simplest forms that could describe the strongly non-monotonous behavior of spectral parameters. There is no deeper reason for this choice, and for the fitting purposes, other functions could be selected if the data points would allow doing so.

## 4. Conclusions

In this work, the possibility of solving the inverse spectral problem for a system with two coupled hydrogen bonds was demonstrated on the example of 61 complexes formed by 4-hydroxypyridine anion with two substituted methanols with OHO and OHN hydrogen bonds. The algorithm for using two-dimensional correlations is as follows: for measured or calculated change of two given spectral parameters, one should find isolines on their corresponding plots and overlap them (see Figure 2). The coordinates of the intersection point of the two isolines will give the proton positions in both hydrogen bonds. It was demonstrated that any pair of parameters Δ*δ*_N4_, Δ*δ*_C1_, Δ*δ*_C26_, Δ*δ*_C35_ and Δ*δ*_H35_ is suitable for the evaluation of hydrogen bond geometries. However, Δ*δ*_C1_, Δ*δ*_C26_, Δ*δ*_C35_ and Δ*δ*_H35_ are more easily available from an experiment.

Thus far, we have only tested our approach computationally on three examples of complexes. The applicability of the method “in practice” awaits experimental verification by future researchers. Indeed, the proposed approach has obvious limitations. Firstly, such 2D maps are constructed for each compound individually (in our case for 4-hydroxypyridine anion). Secondly, the experimental application of the approach requires the measurements of signals that are not averaged out by fast (in the NMR time scale) molecular exchange—a condition that is not necessarily satisfied for intermolecular complexes. The coexistence of several types of molecular complexes in a solution at the same time can also complicate the solving of the reverse spectral problem. Thirdly, the accuracy of the 2D chemical shift maps worsens in the presence of additional non-covalent interactions between the proton donors and the central proton-accepting molecule. Fourthly, for other systems, multiple extrema in the 2D maps of spectral parameters can make determination of hydrogen bonds geometries non-unequivocal, as shown in Figure 7. An unfortunate combination of all four factors could, of course, render the proposed approach totally unreliable. Nevertheless, we have shown in principle that, for systems where these limitations are absent or can be neglected, the NMR spectral data can be sufficient to solve the two-dimensional reverse spectral problem even with a significant entanglement of spectral parameters.

The accuracy of geometry estimations with this approach for systems with two OHO and OHN hydrogen bonds (and without additional interactions) is in the range ±0.04 Å.

## Figures and Tables

**Table 1:** List of substituents of 4-hydroxypyridine anion with two cyano-substituted methanol molecules. Pattern of substitution used for the selection of proton donors in complexes shown in Figure 1. X stands for one of the following substituents: CN, NO_2_, OMe or F.

*R*_1_	*R*_2_	*R*_3_	*R*_4_	*R*_5_	*R*_6_
H	H	H	X	H	H
H	H	H	X	X	H
H	H	H	X	X	X
X	H	H	H	H	H
X	H	H	X	H	H
X	H	H	X	X	H
X	H	H	X	X	X
X	X	H	H	H	H
X	X	H	X	H	H
X	X	H	X	X	H
X	X	H	X	X	X
X	X	X	H	H	H
X	X	X	X	H	H
X	X	X	X	X	H
X	X	X	X	X	X

**Table 2:** Results of testing the proposed approach for three additional complexes, shown in Figure 8: **1** (proton donors CH_3_CHFOH, CF_3_CHFOH), **2** (proton donors CH_3_CHFOH, CF_3_CHFOH), **3** (proton donors CF_3_CHFOH, CH_3_CHFOH).

Complex (Proton Donors)	Calculated Geometry	Pair of Parameters for Prediction	Predicted Geometry
**1** (CH_3_CHFOH, CF_3_CHFOH)	*q*_1a_ = −0.27 Å, *q*_1b_ = −0.20 Å	Δ*δ*_C1_ = 1.2 ppm, Δ*δ*_N4_ = −37.0 ppm	*q*_1a_ = −0.29 Å, *q*_1b_ = −0.21 Å
		Δ*δ*_C26_ = −1.2 ppm, Δ*δ*_C35_ = −5.5 ppm,	*q*_1a_ = −0.30 Å, *q*_1b_ = −0.17 Å
		Δ*δ*_C35_ = −5.5 ppm, Δ*δ*_H35_ = 0.03 ppm	*q*_1a_ = −0.32 Å, *q*_1b_ = −0.19 Å
**2** (CF_3_CHFOH, CF_3_CHFOH)	*q*_1a_ = −0.19 Å, *q*_1b_ = −0.23 Å	Δ*δ*_C1_ = 0.6 ppm, Δ*δ*_N4_ = −29.4 ppm	*q*_1a_ = −0.20 Å, *q*_1b_ = −0.23 Å
		Δ*δ*_C26_ = −2.2 ppm, Δ*δ*_C35_ = −4.6 ppm,	*q*_1a_ = −0.15 Å, *q*_1b_ = −0.20 Å
		Δ*δ*_C35_ = −4.6 ppm, Δ*δ*_H35_ = 0.22 ppm	*q*_1a_ = −0.18 Å, *q*_1b_ = −0.20 Å
**3** (CF_3_CHFOH, CH_3_CHFOH)	*q*_1_a_ = −0.17 Å, *q*_1_b_ = −0.31 Å	Δ*δ*_C1_ = 0.0 ppm, Δ*δ*_N4_ = −14.8 ppm	*q*_1a_ = −0.20 Å, *q*_1b_ = −0.33 Å
		Δ*δ*_C26_ = −3.0 ppm, Δ*δ*_C35_ = −3.0 ppm,	*q*_1a_ = −0.01 Å, *q*_1b_ = −0.28 Å
		Δ*δ*_C35_ = −3.0 ppm, Δ*δ*_H35_ = 0.24 ppm	*q*_1a_ = −0.16 Å, *q*_1b_ = −0.27 Å

## Data Availability

Data is contained within the article or Supplementary Material.

## Supplementary Materials

The following supporting information can be downloaded at: https://www.mdpi.com/article/10.3390/molecules27123923/s1, Table S1:Geometric parameters of OHO and OHN hydrogen bonds; Table S2: Coefficients *a*, *b*_1_, *c*_1_, *d*_1_, *b*_2_, *c*_2_ and *d*_2_ for each discussed spectral parameter, *R*^2^ for each approximation; Figure S1:Δ*δ*_C35_ for complexes of hydroxypyridine anion with a single substituted methanol molecule from an oxygen side (OHO hydrogen bond) Δ*δ*_C35a_ and from a nitrogen site (OHN hydrogen bond) Δ*δ*_C35b_. Distributions of the sum Δ*δ*_C35_ = Δ*δ*_C35a_ + Δ*δ*_C25b_ along *q*_1a_ and *q*_1b_ hydrogen bonds; Figure S2: Δ*δ*_C26_ for complexes of hydroxypyridine with a single substituted methanol molecule from an oxygen side (OHO hydrogen bond) Δ*δ*_C26a_ and from a nitrogen site (OHN hydrogen bond) Δ*δ*_C26b_. Distributions of the sum Δ*δ*_C26__ = Δ*δ*_C26a_ + Δ*δ*_C26b_ along *q*_1a_ and *q*_1b_ hydrogen bonds; Figure S3: Δ*δ*_C26_–Δ*δ*_C26_, Δ*δ*_C35_–Δ*δ*_C35__ along *q*_1a_ and *q*_1b_; Figure S4: Solution of the inverse spectral problem for *R*_1_ = H, *R*_2_ = F, *R*_3_ = CH_3_, *R*_4_ = H, *R*_5_ = F, *R*_6_ = CF_3_; Figure S5: Solution of the inverse spectral problem for *R*_1_ = H, *R*_2_ = F, *R*_3_ = CF_3_, *R*_4_ = H, *R*_5_ = F, *R*_6_ = CF_3_; Figure S6: Solution of the inverse spectral problem for *R*_1_ = H, *R*_2_ = F, *R*_3_ = CF_3_, *R*_4_ = H, *R*_5_ = F, *R*_6_ = CH_3_; Figure S7: Optimized geometry of complex 3 with CF_3_CFOH and CH_3_CFHOH as hydrogen bond donors.

## Sample Availability

Samples of the compounds are not available from the authors.
