# Supplementary material for: Simultaneous Estimation of Two Coupled Hydrogen Bond Geometries from Pairs of Entangled NMR Parameters: The Test Case of 4-Hydroxypyridine Anion
Source: Molecules. 2022 Jun 18;27(12):3923. doi: 10.3390/molecules27123923 (PMC9229270; doi:10.3390/molecules27123923)

*Supporting information for*

# Simultaneous estimation of two coupled hydrogen bond geometries from pairs of entangled NMR parameters: the test case of 4-hydroxypyridine anion


Elena Yu. Tupikina,*[,1] Mark Sigalov,[2] Peter M. Tolstoy[1]

[1] Institute of Chemistry, St. Petersburg State University, St. Petersburg, Russia

[2] Department of Chemistry, Ben-Gurion University of the Negev, Beer-Sheva, Israel


Table of contents


| | | |
|---|---|---|
| **Table S1** | Geometric parameters of OHO and OHN hydrogen bonds | 2 |
| **Table S2** | Coefficients $a$, $b_1$, $c_1$, $d_1$, $b_2$, $c_2$ and $d_2$ for each discussed spectral parameter, $R^2$ for each approximation | 4 |
| **Figure S1** | $\Delta\delta_{C35}$ for complexes of hydroxypyridine anion with a single substituted methanol molecule from an oxygen side (OHO hydrogen bond) $\Delta\delta_{C35a}$ and from a nitrogen site (OHN hydrogen bond) $\Delta\delta_{C35b}$. Distributions of the sum $\Delta\delta_{C35\_\Sigma}$ = $\Delta\delta_{C35a}$ + $\Delta\delta_{C25b}$ along $q_{1a}$ and $q_{1b}$ hydrogen bonds | 5 |
| **Figure S2** | $\Delta\delta_{C26}$ for complexes of hydroxypyridine with a single substituted methanol molecule from an oxygen side (OHO hydrogen bond) $\Delta\delta_{C26a}$ and from a nitrogen site (OHN hydrogen bond) $\Delta\delta_{C26b}$. Distributions of the sum $\Delta\delta_{C26\_\Sigma}$ = $\Delta\delta_{C26a}$ + $\Delta\delta_{C26b}$ along $q_{1a}$ and $q_{1b}$ hydrogen bonds | 6 |
| **Figure S3** | $\Delta\delta_{C26} - \Delta\delta_{C26\_\Sigma}$, $\Delta\delta_{C35} - \Delta\delta_{C35\_\Sigma}$ along $q_{1a}$ and $q_{1b}$ | 7 |
| **Figure S4** | Solution of the inverse spectral problem for $R_1$=H, $R_2$=F, $R_3$=$CH_3$, $R_4$=H, $R_5$=F, $R_6$=$CF_3$ | 8 |
| **Figure S5** | Solution of the inverse spectral problem for $R_1$=H, $R_2$=F, $R_3$=$CF_3$, $R_4$=H, $R_5$=F, $R_6$=$CF_3$ | 9 |
| **Figure S6** | Solution of the inverse spectral problem for $R_1$=H, $R_2$=F, $R_3$=$CF_3$, $R_4$=H, $R_5$=F, $R_6$=$CH_3$ | 10 |
| **Figure S7** | Optimized geometry of complex **3** with $CF_3CFOH$ and $CH_3CFHOH$ as hydrogen bond donors. | 11 |

**Table S1**. Geometric parameters of OHO and OHN hydrogen bonds.

| $R_1$ | $R_2$ | $R_3$ | $R_4$ | $R_5$ | $R_6$ | $r_1$, Å | $r_2$, Å | $r_3$, Å | $r_4$, Å | ∠OHO, ° | ∠NHO, ° |
|---|---|---|---|---|---|---|---|---|---|---|---|
| H | H | H | H | H | H | 1.00 | 1.67 | 1.74 | 1.00 | 169 | 171 |
| H | H | CN | H | H | H | 1.02 | 1.56 | 1.76 | 1.00 | 167 | 171 |
| H | CN | CN | H | H | H | 1.06 | 1.44 | 1.78 | 1.00 | 169 | 171 |
| CN | CN | CN | H | H | H | 1.49 | 1.04 | 1.81 | 0.99 | 178 | 169 |
| H | H | H | CN | H | H | 1.00 | 1.68 | 1.61 | 1.03 | 168 | 168 |
| H | H | CN | CN | H | H | 1.02 | 1.59 | 1.64 | 1.02 | 166 | 168 |
| H | CN | CN | CN | H | H | 1.05 | 1.46 | 1.66 | 1.01 | 168 | 168 |
| CN | CN | CN | CN | H | H | 1.47 | 1.04 | 1.70 | 1.01 | 177 | 166 |
| H | H | H | CN | CN | H | 1.00 | 1.70 | 1.48 | 1.07 | 168 | 170 |
| H | H | CN | CN | CN | H | 1.01 | 1.59 | 1.51 | 1.06 | 167 | 170 |
| H | CN | CN | CN | CN | H | 1.04 | 1.48 | 1.55 | 1.04 | 167 | 168 |
| CN | CN | CN | CN | CN | H | 1.44 | 1.05 | 1.61 | 1.03 | 176 | 167 |
| H | H | H | CN | CN | CN | 0.99 | 1.74 | 1.07 | 1.54 | 167 | 174 |
| H | H | CN | CN | CN | CN | 1.00 | 1.64 | 1.09 | 1.49 | 165 | 178 |
| H | CN | CN | CN | CN | CN | 1.02 | 1.53 | 1.09 | 1.50 | 167 | 172 |
| CN | CN | CN | CN | CN | CN | 1.09 | 1.37 | 1.11 | 1.45 | 171 | 178 |
| H | H | $NO_2$ | H | H | H | 1.04 | 1.50 | 1.76 | 1.00 | 168 | 171 |
| H | $NO_2$ | $NO_2$ | H | H | H | 1.52 | 1.02 | 1.81 | 0.99 | 178 | 169 |
| $NO_2$ | $NO_2$ | $NO_2$ | H | H | H | 1.68 | 0.99 | 1.82 | 0.99 | 176 | 168 |
| H | H | H | $NO_2$ | H | H | 1.00 | 1.68 | 1.54 | 1.05 | 168 | 171 |
| H | H | $NO_2$ | $NO_2$ | H | H | 1.03 | 1.52 | 1.57 | 1.04 | 168 | 171 |
| H | $NO_2$ | $NO_2$ | $NO_2$ | H | H | 1.51 | 1.03 | 1.64 | 1.02 | 177 | 170 |
| $NO_2$ | $NO_2$ | $NO_2$ | $NO_2$ | H | H | 1.68 | 1.00 | 1.65 | 1.01 | 176 | 170 |
| H | H | H | $NO_2$ | $NO_2$ | H | 0.99 | 1.73 | 1.05 | 1.66 | 167 | 166 |
| H | H | $NO_2$ | $NO_2$ | $NO_2$ | H | 1.02 | 1.54 | 1.05 | 1.64 | 171 | 167 |
| H | $NO_2$ | $NO_2$ | $NO_2$ | $NO_2$ | H | 1.39 | 1.07 | 1.07 | 1.55 | 179 | 169 |
| $NO_2$ | $NO_2$ | $NO_2$ | $NO_2$ | $NO_2$ | H | 1.65 | 1.00 | 1.45 | 1.08 | 176 | 175 |
| H | H | H | $NO_2$ | $NO_2$ | $NO_2$ | 0.99 | 1.75 | 1.03 | 1.77 | 166 | 156 |
| H | H | $NO_2$ | $NO_2$ | $NO_2$ | $NO_2$ | 1.01 | 1.57 | 1.04 | 1.73 | 171 | 162 |
| H | $NO_2$ | $NO_2$ | $NO_2$ | $NO_2$ | $NO_2$ | 1.08 | 1.38 | 1.04 | 1.68 | 177 | 164 |
| $NO_2$ | $NO_2$ | $NO_2$ | $NO_2$ | $NO_2$ | $NO_2$ | 1.60 | 1.01 | 1.06 | 1.62 | 167 | 165 |
| H | OMe | OMe | H | OMe | OMe | 1.02 | 1.55 | 1.62 | 1.02 | 171 | 174 |
| H | OMe | OMe | H | H | OMe | 1.03 | 1.53 | 1.74 | 1.00 | 172 | 168 |
| H | OMe | OMe | H | H | H | 1.03 | 1.52 | 1.76 | 1.00 | 172 | 171 |
| H | OMe | OMe | OMe | OMe | OMe | 1.03 | 1.52 | 1.65 | 1.02 | 172 | 176 |
| H | H | OMe | H | OMe | OMe | 1.00 | 1.69 | 1.68 | 1.02 | 162 | 163 |
| H | H | OMe | H | H | H | 1.01 | 1.63 | 1.74 | 1.00 | 170 | 168 |
| H | H | OMe | H | H | H | 1.01 | 1.60 | 1.75 | 1.00 | 168 | 171 |
| H | H | OMe | OMe | OMe | OMe | 1.00 | 1.66 | 1.13 | 1.40 | 167 | 175 |
| H | H | H | H | H | OMe | 1.00 | 1.68 | 1.61 | 1.03 | 168 | 174 |
| H | H | H | H | H | OMe | 1.00 | 1.67 | 1.65 | 1.02 | 169 | 172 |
| H | H | H | OMe | OMe | OMe | 0.99 | 1.71 | 1.13 | 1.41 | 167 | 175 |
| OMe | OMe | OMe | H | H | OMe | 1.05 | 1.48 | 1.74 | 1.00 | 170 | 168 |
| OMe | OMe | OMe | OMe | OMe | OMe | 1.03 | 1.53 | 1.13 | 1.40 | 170 | 178 |

| | | | | | | | | | | | |
|---|---|---|---|---|---|---|---|---|---|---|---|
| OMe | OMe | OMe | H | OMe | OMe | 1.04 | 1.49 | 1.62 | 1.02 | 156 | 172 |
| OMe | OMe | OMe | H | H | H | 1.05 | 1.46 | 1.75 | 1.00 | 170 | 171 |
| H | F | F | H | H | H | 1.41 | 1.07 | 1.80 | 0.99 | 179 | 169 |
| H | F | F | H | H | F | 1.08 | 1.38 | 1.64 | 1.02 | 177 | 171 |
| H | F | F | H | H | F | 1.06 | 1.42 | 1.51 | 1.06 | 176 | 176 |
| H | F | F | F | F | F | 1.03 | 1.49 | 1.07 | 1.54 | 175 | 176 |
| H | H | F | H | H | H | 1.03 | 1.54 | 1.75 | 1.00 | 171 | 171 |
| H | H | F | H | H | F | 1.02 | 1.55 | 1.62 | 1.02 | 171 | 171 |
| H | H | F | H | F | F | 1.02 | 1.57 | 1.47 | 1.08 | 171 | 177 |
| H | H | F | F | F | F | 1.01 | 1.62 | 1.07 | 1.56 | 170 | 175 |
| F | F | F | H | H | H | 1.53 | 1.02 | 1.80 | 0.99 | 175 | 169 |
| F | F | F | H | H | F | 1.52 | 1.02 | 1.68 | 1.01 | 175 | 169 |
| F | F | F | H | F | F | 1.50 | 1.03 | 1.58 | 1.03 | 175 | 174 |
| F | F | F | F | F | F | 1.43 | 1.05 | 1.09 | 1.47 | 176 | 176 |
| H | H | H | H | H | F | 1.00 | 1.67 | 1.60 | 1.03 | 168 | 172 |
| H | H | H | H | F | F | 1.00 | 1.69 | 1.41 | 1.11 | 168 | 177 |
| H | H | H | F | F | F | 0.99 | 1.73 | 1.06 | 1.58 | 167 | 175 |

**Table S2**. Approximation coefficients for $\Delta f(q_{1a}, q_{1b}) = a + b_1 q_{1a} + c_1 q_{1a}^2 + d_1 q_{1a}^3 + b_2 q_{1b} + c_2 q_{1b}^2 + d_2 q_{1b}^3$ (for $q_{1a}$ and $q_{1b}$ in Å, $\Delta f$ in ppm) and $R^2$.

| $\Delta f$ | $a$ | $b_1$ | $c_1$ | $d_1$ | $b_2$ | $c_2$ | $d_2$ | $R^2$ |
|---|---|---|---|---|---|---|---|---|
| $\Delta\delta_{C1}$ | –1.1 | –15.35 | –9.15 | 30.84 | 2.18 | –2.33 | 9.91 | 0.97 |
| $\Delta\delta_{N4}$ | –49.4 | 41.45 | 4.37 | –8.12 | –106.3 | 14.95 | –133.9 | 0.99 |
| $\Delta\delta_{H1\_HB}$ | 17.0 | 4.12 | –87.91 | –90.03 | 0.21 | –6.23 | –16.56 | 0.93 |
| $\Delta\delta_{H2\_HB}$ | 17.9 | 1.38 | –2.96 | –33.24 | 5.13 | –79.22 | –85.15 | 0.95 |
| $\Delta\delta_{C26}$ | –3.0 | –4.03 | 0.81 | –9.606 | –2.07 | 0.67 | 24.56 | 0.82 |
| $\Delta\delta_{C35}$ | –8.1 | 1.10 | –1.37 | 23.99 | –16.86 | 13.97 | 30.38 | 0.98 |
| $\Delta\delta_{H35}$ | 0.4 | 0.84 | –0.65 | –0.37 | 0.35 | 1.12 | 0.49 | 0.86 |

**Figure S1**. Distribution of the chemical shift change upon complexation $\Delta\delta_{C35}$ for complexes of hydroxypyridine with a single substituted methanol molecule (a) from the oxygen side (OHO hydrogen bond) $\Delta\delta_{C35a}$, (b) from the nitrogen site (OHN hydrogen bond) $\Delta\delta_{C35b}$. (c) Distributions of the sum of the changes upon formation of both OHO and OHN hydrogen bonds $\Delta\delta_{C35\_\Sigma} = \Delta\delta_{C35a} + \Delta\delta_{C35b}$ along $q_1$ coordinates for OHO ($q_{1a}$) and OHN ($q_{1b}$) hydrogen bonds. Coefficients $a_1$, $b_1$, $c_1$, $d_1$, $a_2$, $b_2$, $c_2$ are given near each plot. Isolines drawn with a step of 0.5 ppm.

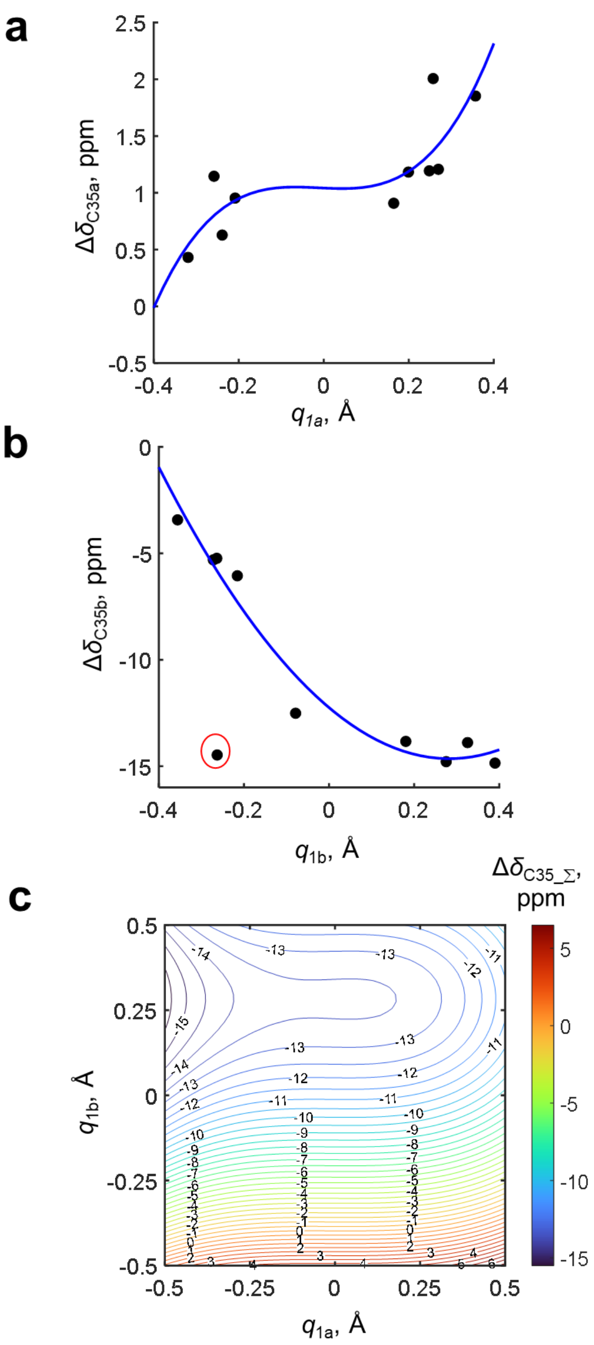


$$\Delta\delta_{C35a} = 19.25 \cdot q_{1a}^3 + 0.69 \cdot q_{1a}^2 - 0.17 \cdot q_{1a} + 1.04$$

$$\Delta\delta_{C35b} = 1.52 \cdot q_{1b}^3 + 29.11 \cdot q_{1b}^2 - 16.83 \cdot q_{1b} - 12.25$$

$$\Delta\delta_{C35\_\Sigma} = \Delta\delta_{C35a} + \Delta\delta_{C35b}$$

**Figure S2**. Distribution of the chemical shift change upon complexation $\Delta\delta_{C26}$ for complexes of hydroxypyridine with a single substituted methanol molecule (a) from the oxygen side (OHO hydrogen bond) $\Delta\delta_{C26a}$, (b) from the nitrogen site (OHN hydrogen bond) $\Delta\delta_{C26b}$. (c) Distributions of the sum of the changes upon formation of both OHO and OHN hydrogen bonds $\Delta\delta_{C26\_\Sigma} = \Delta\delta_{C26a} + \Delta\delta_{C26b}$ along $q_1$ coordinates for OHO ($q_{1a}$) and OHN ($q_{1b}$) hydrogen bonds. Coefficients $a_1$, $b_1$, $c_1$, $d_1$, $a_2$, $b_2$, $c_2$ are given near each plot. Isolines drawn with a step of 0.5 ppm.

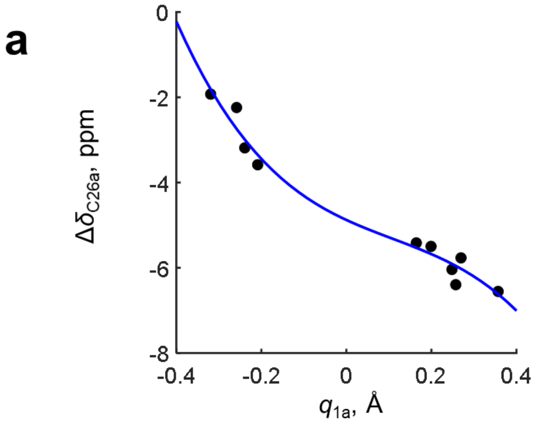


$$\Delta\delta_{C26a} = -24.15 \cdot q_{1a}^3 + 7.91 \cdot q_{1a}^2 - 4.62 \cdot q_{1a} - 4.88$$

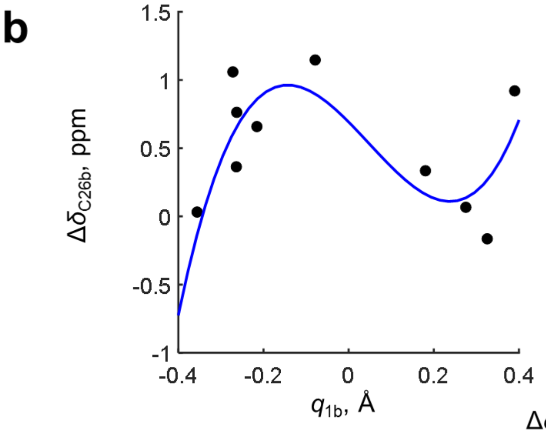


$$\Delta\delta_{C26b} = 30.87 \cdot q_{1b}^3 - 4.38 \cdot q_{1b}^2 - 3.15 \cdot q_{1b} + 0.69$$

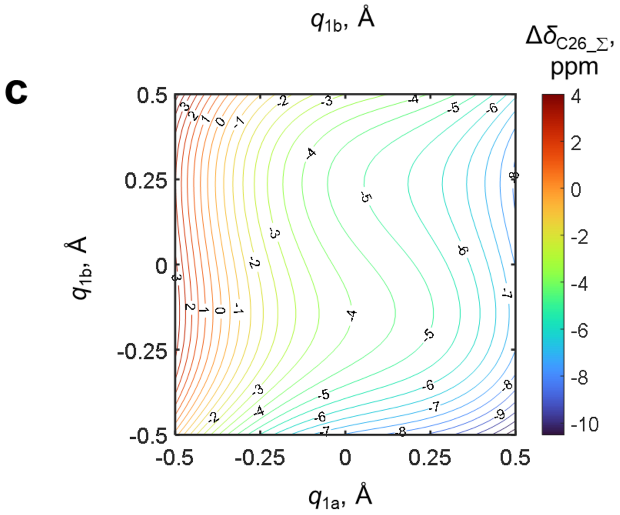


$$\Delta\delta_{C26\_\Sigma} = \Delta\delta_{C26a} + \Delta\delta_{C26b}$$

## Non-additivity (cooperativity) of hydrogen bonds formed by hydroxypyridune anion

**Figure S3**. Difference (a) between carbon chemical shift changes upon formation of two hydrogen bond simultaneously $\Delta\delta_{C26}$ and of the sum of a change upon formation of OHO and OHN hydrogen bonds $\Delta\delta_{C26\_\Sigma} = \Delta\delta_{C26a} + \Delta\delta_{C26b}$, (b) between carbon chemical shift changes upon formation of two hydrogen bond simultaneously $\Delta\delta_{C35}$ and of the sum of a change upon formation of OHO and OHN hydrogen bonds $\Delta\delta_{C35\_\Sigma} = \Delta\delta_{C35a} + \Delta\delta_{C35b}$ along $q_1$ coordinates for OHO ($q_{1a}$) and OHN ($q_{1b}$) hydrogen bonds. Isolines drawn with a step of 0.2 ppm, and 0.5 ppm respectively.

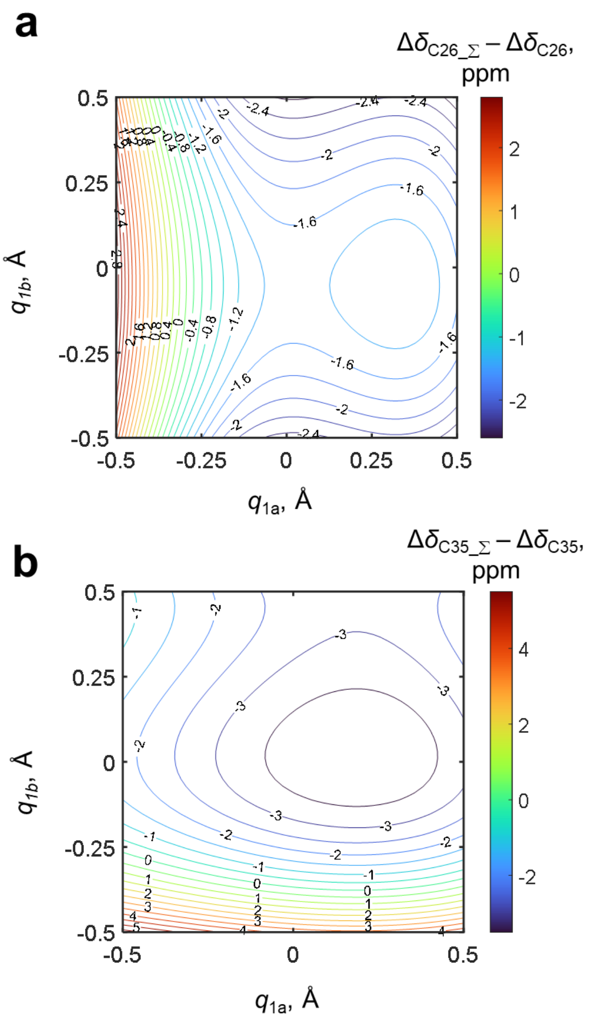

**Figure S4**. Solution of inverse spectral problem for $R_1$=H, $R_2$=F, $R_3$=$CH_3$, $R_4$=H, $R_5$=F, $R_6$=$CF_3$ based on a following pairs of spectral parameters (a) $\Delta\delta_{C1}$ and $\Delta\delta_{N4}$, (b) $\Delta\delta_{C26}$ and $\Delta\delta_{C35}$, (c) $\Delta\delta_{C35}$ and $\Delta\delta_{H35}$. Green dots correspond to predicted values of $q_{1a}$ and $q_{1b}$, black dots correspond to calculated ones.

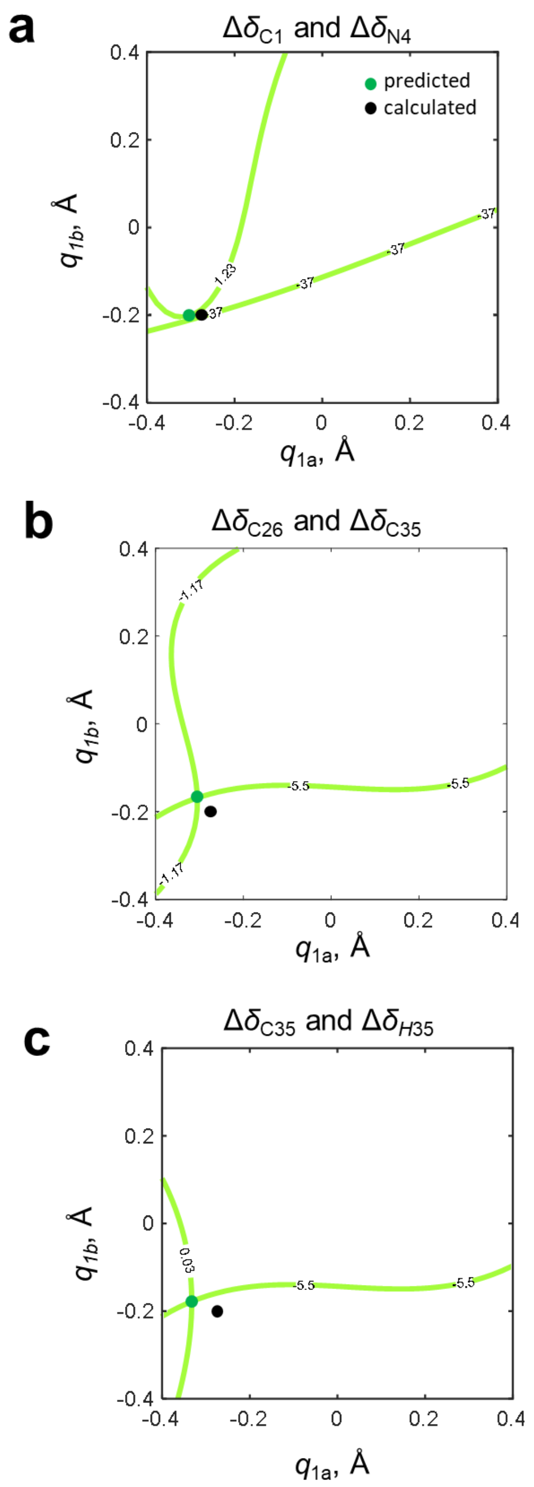

**Figure S5**. Solution of inverse spectral problem for $R_1$=H, $R_2$=F, $R_3$=$CF_3$, $R_4$=H, $R_5$=F, $R_6$=$CF_3$ based on a following pairs of spectral parameters (a) $\Delta\delta_{C1}$ and $\Delta\delta_{N4}$, (b) $\Delta\delta_{C26}$ and $\Delta\delta_{C35}$, (c) $\Delta\delta_{C35}$ and $\Delta\delta_{H35}$. Green dots correspond to predicted values of $q_{1a}$ and $q_{1b}$, black dots correspond to calculated ones.

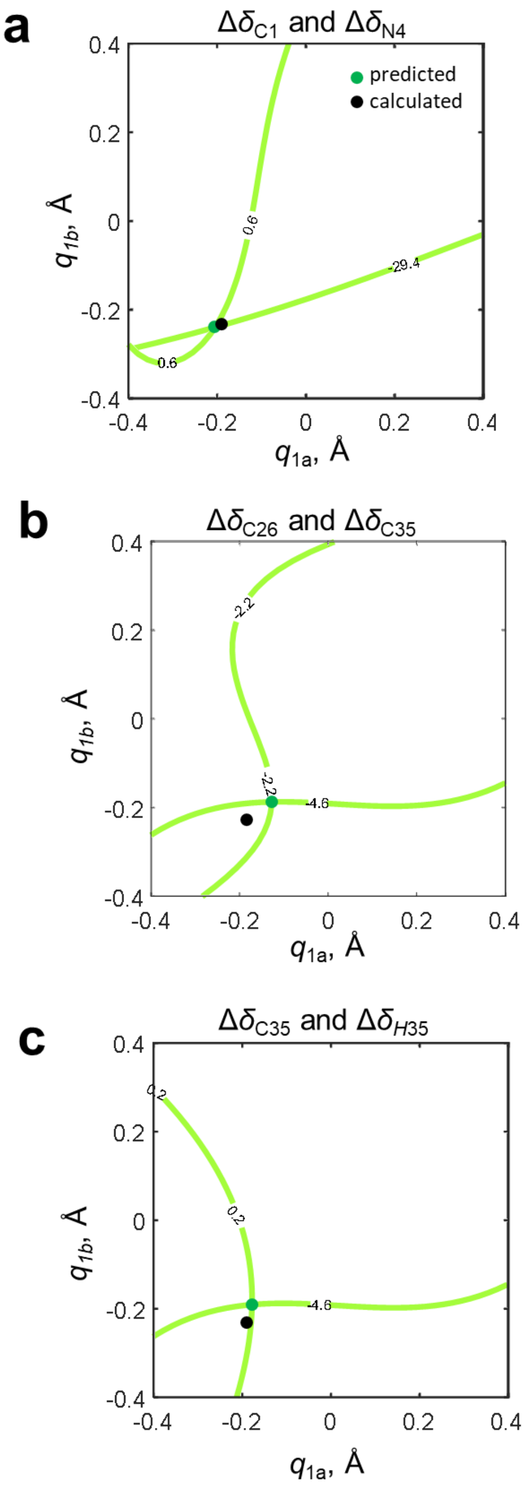

**Figure S6**. Solution of inverse spectral problem for $R_1$=H, $R_2$=F, $R_3$=$CF_3$, $R_4$=H, $R_5$=F, $R_6$=$CH_3$ based on a following pairs of spectral parameters (a) $\Delta\delta_{C1}$ and $\Delta\delta_{N4}$, (b) $\Delta\delta_{C26}$ and $\Delta\delta_{C35}$, (c) $\Delta\delta_{C35}$ and $\Delta\delta_{H35}$. Green dots correspond to predicted values of $q_{1a}$ and $q_{1b}$, black dots correspond to calculated ones.

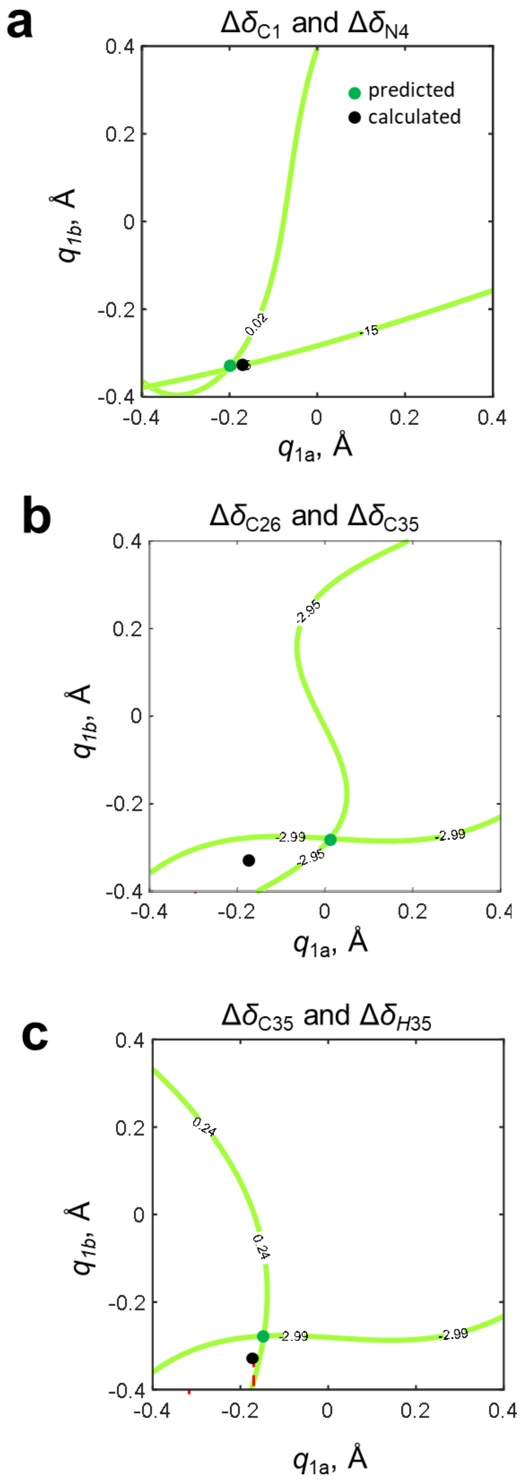

**Figure S7**. Optimized geometry of complex **3** with $CF_3CFOH$ and $CH_3CFHOH$ as hydrogen bond donors.

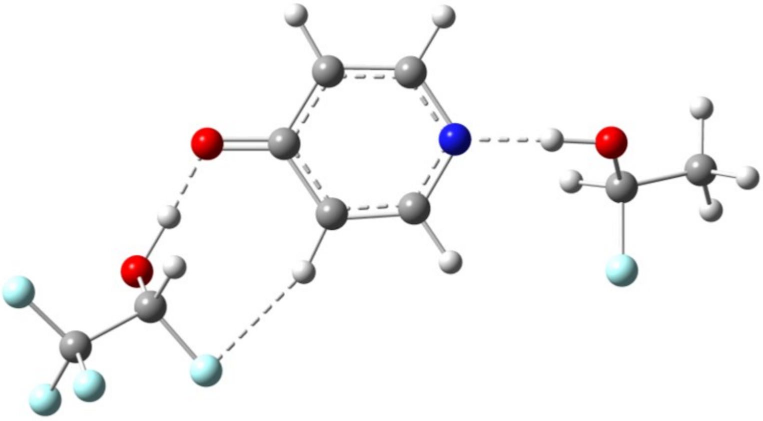

Supplement: Supplementary file 1 [file molecules-27-03923-s001.zip › molecules-1719992-supplementary.pdf]
